# Standardizing Zebrafish Behavioral Paradigms Across Life Stages: An Effort Towards Translational Pharmacology

**DOI:** 10.3389/fphar.2022.833227

**Published:** 2022-01-20

**Authors:** Barbara Dutra Petersen, Kanandra Taisa Bertoncello, Carla Denise Bonan

**Affiliations:** ^1^ Programa de Pós-Graduação em Medicina e Ciências da Saúde, Escola de Medicina, Pontifícia Universidade Católica do Rio Grande do Sul, Porto Alegre, Brazil; ^2^ Laboratório de Neuroquímica e Psicofarmacologia, Escola de Ciências da Saúde e da Vida, Pontifícia Universidade Católica do Rio Grande do Sul, Porto Alegre, Brazil; ^3^ Programa de Pós-Graduação em Biologia Celular e Molecular, Escola de Ciências da Saúde e da Vida, Pontifícia Universidade Católica do Rio Grande do Sul, Porto Alegre, Brazil

**Keywords:** zebrafish, behavioral tests, ontology, anxiety, exploratory behavior, sociability, cognition (Min. 5–Max. 8)

## Abstract

Zebrafish is a prominent vertebrate model, with many of its advantages related to its development, life cycle, and translational ability. While a great number of behavioral phenotypes and tasks to evaluate them are available, longitudinal studies across zebrafish life stages are scarce and made challenging because of the differences between protocols and endpoints assessed at each life stage. In this mini review, we highlight the relevance that longitudinal studies could have for neurobehavioral pharmacology using this model. We also present possible strategies to standardize behavior endpoints in domains related to human diseases throughout the life cycle, especially between larvae and adult fish. Furthermore, we discuss the remaining difficulties of these analyses and explore future advances needed to bridge this knowledge gap.

## 1 Introduction

Zebrafish is a widely used vertebrate model species in research fields such as genetics, pharmacology, developmental biology, neuroscience, among others (Senger et al., 2004; Egan et al., 2009; Spence et al., 2011; Siebel et al., 2015). As an alternative to rodents, zebrafish has numerous advantages. Their habits are diurnal, they reproduce throughout the year and produce a large number of eggs with each reproduction (Kalueff et al., 2014). Furthermore, external development and the absence of parental care reduce the epigenetic influences of parental animals (Mushtaq et al., 2013) and allow manipulations during embryonic development (Kalueff et al., 2014). Initial development is rapid - embryogenesis lasts approximately 24 h (Dahm et al., 2006) - with most neurochemical pathways already functional, although immature, at around 60 h post-fertilization (hpf) (Arenzana et al., 2005; Sallinen et al., 2009; Cocco et al., 2017; Monesson-Olson et al., 2018).

The high capacity of behavioral analysis is also a feature of this model. The importance of behavioral traits in research stems mostly from it being a functional readout of neural activity (Gerlai et al., 2000; Valente et al., 2012; Orger and De Polavieja, 2017). In this sense, although zebrafish and mammals have marked anatomical differences in the structural organization of the central nervous system (CNS) (Wullimann and Mueller, 2004; Sager et al., 2010), there is a strong functional homology, reflected in the similarity of effects in response to exposure to compounds between zebrafish and mammals (Bailey et al., 2013) and in the successful adaptation of many behavioral paradigms of rodents to this species (Kalueff et al., 2014). In zebrafish, behavioral assessments can be performed as early as 17 hpf, still inside the eggs (Kokel et al., 2010), and more complex tasks are available when the animals reach a state of free-swimming and active search for food within 5 days after fertilization (dpf) (Neuhauss, 2003). As fish develops, behaviors increase in complexity. Most studies focus on either larval or adult stages, with around 190 distinct behavioral phenotypes already described for zebrafish (Kalueff et al., 2013). These phenotypes combined can be arranged in behavioral domains (Kalueff et al., 2013; Kalueff et al., 2014) and serve as significant endpoints in biomedical and human disease translational research (Ninkovic and Bally-Cuif, 2006; Kalueff et al., 2014; Fontana et al., 2018), toxicology, and pharmacology (Goldsmith, 2004; Bailey et al., 2013; MacRae and Peterson, 2015; Costa et al., 2020).

Granting all this, translationality of behavior across life stages in zebrafish is still poorly explored, mainly because paradigms for larvae and adult fish differ in form and endpoints, thus not allowing direct comparisons between stages. In this review, we will discuss the significance of this gap focusing on behavioral responses to pharmacological interventions, the currently used paradigms to each life stage and behavioral domain, the alternative paradigms that can potentially be used across stages to close this gap, and the drawbacks and benefits of each strategy.

## 2 Variability of Pharmacological Responses by Life Stages

Age is a factor that might influence zebrafish behavior and its pharmacological responses, especially in early life stages when results reflect specific time windows in which the neural systems are not yet mature (Orger and De Polavieja, 2017). Despite the difficulties for transposing results across ages, posed by the variations of endpoints, studies of chemicals in varying developmental stages indicate the advantages zebrafish could have if this gap was diminished. One drug that was extensively studied throughout zebrafish development is the NMDA receptor antagonist MK-801. In general, neurobehavioral actions of this drug are conserved in zebrafish (Chen et al., 2010; Sison and Gerlai, 2011), and the findings are comparable to those previously described in rodents (Löscher and Hönack, 1992). Some studies using MK-801 demonstrate different behavioral responses of zebrafish during its development.

Age-related locomotor responses to MK-801 were reported in zebrafish (Menezes et al., 2015). In this study, the locomotor response of zebrafish to MK-801 through the development, from 30 dpf to 2 years post-fertilization (ypf) was evaluated. Distance traveled showed that 30-day-old zebrafish did not respond to MK-801, whereas in animals aged 60 dpf, 120 dpf, and 2 ypf, the antagonist promoted an increase in locomotor activity at the concentration of 5 µM. When the NMDA receptor subunit gene expression was analyzed through the development (7 dpf–2 ypf), the results showed variations in the expression of NR1.1 and NR2A.2 and NR2C.1 subunits throughout the development. With this, the changes in locomotor responses to MK-801 exposure through the development could be a consequence of differential NMDA receptor subunit expression (Menezes et al., 2015). This result of developmental responses to MK-801 highlights the significance of cross-ages studies, and how this model can uncover characteristics of neural development useful for pharmacological interventions.

The effects of MK-801 were assessed in more complex behaviors. Social interactions variations in response to MK-801 demonstrated that zebrafish submitted to a preference/interaction assay show a social behavior that starts to emerge by 2 weeks post-fertilization (wpf) and is robust at 3 wpf (Dreosti et al., 2015). At this point (3 wpf), acute treatment with 100 µM MK-801 disrupted social preference, as was previously shown in adults (Sison and Gerlai, 2011). This suggests that blocking NMDA receptors interferes with the circuitry required for social interactions, both in larva and in adult fish. Cognition, as tested with novel object recognition paradigms, was also evaluated both in larvae and adult zebrafish, demonstrating different responses to MK-801. While in larvae MK-801 did not affect memory retrieval in short- or long-term memory assay in zebrafish at 10 dpf (Andersson et al., 2015), MK-801 impaired object location memory in adult zebrafish on a novel object recognition test (Gaspary et al., 2018). It indicates dependent differences in the action of the antagonist according to age, once in zebrafish larvae MK-801 did not influence the retrieval of memories obtained before the treatment, as was expected by previous adult outcomes.

The studies described here serve as an example and highlight the importance of a better understanding of behavioral phenotypes at different stages of zebrafish development. Results to MK-801 vary depending on age, the complexity of behavior, and endpoint assessed. Therefore, assessing equivalent endpoints throughout life can potentially bring zebrafish pharmacology to a new level. It may facilitate the observations of how responses develop and increase zebrafish potential as a translational model that enables the study of ontogenic changes of molecular drug targets.

## 3 Zebrafish Behavioral Paradigms

### 3.1 General Behavior and Anxiety

General behavior and anxiety in zebrafish are generally evaluated together, although specific tests to evaluate anxiety are available. Regarding the general well-being of zebrafish, this assessment can be performed during behavioral tasks, with the main parameters common to the various tasks being total distance traveled, erratic movements (usually measured by the angle of rotation), and immobility (Westerfield, 2000; Kalueff and Stewart, 2012; Kalueff et al., 2013). The animal’s body position can be observed during these tests and indicate disease or exposure responses to compounds (Kalueff et al., 2013). The assessment of these parameters together can lead to the characterization of complex behaviors such as hypolocomotion, hyperlocomotion, sickness behavior, motor incoordination, among others (Kalueff et al., 2013).

Although these behavioral phenotypes can be evaluated in a wide variety of tests, the general aspects of zebrafish locomotion are usually analyzed in the novel tank or open field tests (Bridi et al., 2017; Nabinger et al., 2020; Petersen et al., 2021). In adults, novel tank and open tank tests are performed in a rectangular or trapezoid aquarium and the typical zebrafish behavior is to stay at the bottom of the apparatus for the first few minutes of testing, followed by top exploration, a phenomenon called bottom-dwelling (Kalueff et al., 2013). Animals also tend to exhibit thigmotaxis, that is, to remain close to the walls of the apparatus, although this is rarely assessed by the difficulty of measuring time at the bottom/top and thigmotaxis simultaneously with a single camera (Kalueff and Stewart, 2012). In larvae, the paradigm used to assess exploration is usually carried out with the camera below or above 24 or 96-well plates, so that thigmotaxis, but not depth preference, is assessed (Creton, 2009). In both larvae and adults, locomotion in these scenarios follows a sinusoidal oscillatory pattern, with alternating phases of higher and lower velocities with a frequency of approximately 5 min (Kalueff and Stewart, 2012; Kalueff et al., 2013).

With this, the main differences in the above-mentioned paradigms for larvae and adults are the positioning of the camera, which impact the endpoints that can be retrieved later with automated analysis software (bottom-dwelling or thigmotaxis), and the form of the apparatus (rectangular or round). Although unusual, the rectangular novel tank test has recently been adapted for older larvae (Golla et al., 2020). In this variation, fish were filmed in groups, and bottom dwelling was measured not by time but by ratio. Despite this, the protocol can be used as an alternative to access larvae in a similar way to adult fish. The main con of this proposal would be to lose the assessment of thigmotaxis.

Another alternative regarding standardization of general behavior across life stages could be filming adult fish from above, in round tanks. This alternative is especially promising for pharmacological studies, once it allows coupling with the Light Motor Response (LMR) test and reduces the number of animals used. The LMR test, also called motor response to light/dark transitions, is widely used in zebrafish larvae, mainly due to its high sensitivity to neuroactive compounds (Emran et al., 2008; Legradi et al., 2014; Klüver et al., 2015; Leuthold et al., 2019), and has already been adapted for adult animals (Shao et al., 2017). For the LMR, after habituation to the tank or well, light condition is changed periodically from light to darkness and behavior is recorded with an infrared enabled camera, with different number and length of cycles described in the literature (Emran et al., 2008; Legradi et al., 2014; Klüver et al., 2015; Leuthold et al., 2019). Alike the novel/open tank, the parameters retrieved are distance traveled, erratic movements, thigmotaxis, immobility, and more, only the analysis is coupled by light condition, hence there are typical behavioral changes exhibited by animals in dark and light environments, and in the transitions between these two conditions.

The three paradigms discussed (rectangular novel tank, round novel tank, and LMR) can also be used to evaluate anxiety-like states in zebrafish. Stress and anxiety models are widely used in research related to neuroscience and as a behavioral parameter for drug discovery studies with effects on the CNS (Kysil et al., 2017). In other experimental models, but especially in zebrafish, the behavioral phenotypes of fear, stress, and anxiety are confusing, and species-specific patterns that help in their differentiation are not well established (Maximino et al., 2010a). Thus, we will use the term “anxiety” to describe the set of these phenotypes. In the novel tank test, anxiety is assessed together with exploratory behavior, by observing the parameters: the latency to swim to the upper half of the tank, the number of transitions between the lower and upper half, the time in each of these zones, the instances of erratic movements and immobility and freezing responses (Maximino et al., 2010a; Kysil et al., 2017). Finally, regarding outcomes that can be evaluated in non-specific paradigms for anxiety, thigmotaxis (time, frequency, crossings between center and borders of the well) is used as an parameter for indicating anxiety in larvae, and in some tests used to assess cognition based on aversive stimuli and also on light transition tests (Maximino et al., 2010a; Schnörr et al., 2012).

Among the specific tests for anxiety, the most recognized is the light/dark preference test, based on the animals’ natural scototaxis. This test has already been adapted for different stages of development and presented with some methodological variations (Serra et al., 1999; Maximino et al., 2010b; Stewart et al., 2011; Golla et al., 2020). In general, the paradigm consists of placing the animal individually in an apparatus with a zone with white walls and floor and another zone with black walls and floor. The parameters used to measure anxiety are number of transitions between zones, latency for the aversive zone, number of risk assessment episodes (rapid entries, without permanence in the aversive zone), thigmotaxis in the aversive zone, time in the aversive zone, number of freezing episodes and erratic movements (Maximino et al., 2010b; Steenbergen et al., 2011; Kysil et al., 2017). The main variation in responses to being placed in this half-black, half-white tank between life stages is that while animals in the larval stage prefer light zones, juvenile and adult animals prefer dark zones (Kalueff et al., 2013; Kysil et al., 2017). Exactly when this transition of preference occurs is still an unknown point. Thus, to enable comparisons between life stages, the researcher must be aware of the preference the animals exhibit and evaluate the outcomes about the zone that the animal tends to avoid (light for adults, dark for larvae) or prefer. Another point of care is the variation of the paradigm that will be used, depending on the age and size of animals. Given that the size of the tank should be adapted to the animal’s size (Buske and Gerlai, 2011) when using smaller animals such as young larvae, the use of sliding doors may be non-practical, thus the paradigm of Serra et al. (1999) should be preferred.

### 3.2 Cognition

The cognition domain comprises the processes involved in learning and memory. The diversity of behavioral paradigms aimed to study this domain in zebrafish is high, however, the most used tasks are based on conditioning to aversive or rewarding stimuli, or based on the natural preference of these animals for new objects or situations (Blank et al., 2009; Cognato et al., 2012; Gaspary et al., 2018; Yashina et al., 2019). Concerning to the tasks based on classical or operant conditioning, the main stimuli are usually aversive, most notably the use of electric shocks (Blank et al., 2009; Hinz et al., 2013; Harmon et al., 2017; Yashina et al., 2019). This type of test is usually performed in multiple sessions and generates long-term memories, and the main outcomes measured are permanence in the zone considered safe or the latency to enter the zone where the animal previously received the aversive stimulus (Blank et al., 2009; Valente et al., 2012; Yashina et al., 2019).

How early zebrafish can learn and retrieve memories is still a point of debate. Classical conditioning tasks have been successfully performed in animals in the early stages of development at 6 dpf (Aizenberg and Schuman, 2011; Harmon et al., 2017) and ∼ 3 wpf (Valente et al., 2012; Matsuda et al., 2017). Regarding operant conditioning, Yashina et al. (2019) tested animals at 7, 14, and 21 dpf, demonstrating that learning varies according to animal size at 7 and 14 dpf, suggesting that body length, not age in days, reflects the brain development necessary for learning in this age group. The duration of memories acquired through conditioning protocols also seems to depend on the test used and the age of the animals (Roberts et al., 2013; Yashina et al., 2019). For short-term memories, the Y-maze is used in adult animals (Cognato et al., 2012; Fontana et al., 2021; Petersen et al., 2021). This paradigm is based on visual cues and the preference of zebrafish for novel situations to form a spatial memory of animals (Cognato et al., 2012). The Y-Maze has not yet been characterized in animals in their early stages of development; however, a paradigm based on visual cues and the presence of a new object in 10 dpf animals was characterized, demonstrating that older larvae can learn spatial tasks (Andersson et al., 2015).

In such a case, studying cognitive alterations across zebrafish development is still a challenge. Although classical conditioning appears to be the safest option, results in young larvae are inconsistent depending on the size of animals, inter-trial interval, and specific paradigm (Valente et al., 2012; Harmon et al., 2017; Yashina et al., 2019). Given the importance of cognitive symptoms in many health conditions, the adaptation and validation of a paradigm suited for such purposes present as major gap in the behavioral neuroscience of zebrafish.

### 3.3 Sociability

The social behavior of zebrafish encompasses the interaction with conspecifics, aggression, and sexual behaviors (Kalueff et al., 2013), all of which are highly conserved in this species (Scott and Sloman, 2004; Stednitz and Washbourne, 2020). So far, studies related to this domain have focused on non-sexual interaction and aggressiveness (Buske and Gerlai, 2011). This behavioral domain starts to develop a little further in development. Non-sexual social interaction encompasses a set of behaviors that develop from the preference for conspecifics at around 3 wpf, and around 45 dpf culminates in ordered swimming in groups (known as shoaling) (Engeszer et al., 2007; Buske and Gerlai, 2011; Dreosti et al., 2015; Stednitz and Washbourne, 2020). The main test for the evaluation of this characteristic is the social interaction test, where the animals are exposed to the vision of conspecifics and their proximity and distance from the zone close to them is evaluated. Factors that influence the results and may be crucial in the observed age differences are the number of fish tested and stimulus fish used, the pairing or not of the size between test fish and stimulus fish, and the living conditions of the animals before the test (Dreosti et al., 2015; Groneberg et al., 2020; Stednitz and Washbourne, 2020). With regard to aggressive behavior, using the tilted mirror and dyadic struggles paradigms, respectively, Carreño Gutiérrez et al. (2018) and Ricci et al. (2013) found results suggesting that the onset of aggressive behavior occurs around 28 dpf.

The current data suggest that social behaviors in zebrafish can be evaluated only at early juvenile stages. It is unknown, however, if more subtle indications of sociability exist and can be explored earlier on. Nonetheless, with the current tests available, the social interaction/preference and the tilted mirror aggression paradigms depend less on group behavior than the shoaling and dyadic fight variations, as well as social preference responses develop earlier than the shoaling responses.

## 4 Conclusion and Future Perspectives

Since the 1970s, when zebrafish started to be used as an animal model, advances have been made in decoupling interfering variables in studies. Some examples of this are the relatively recent availability of isogenic strains (Lange et al., 2013; Kalueff et al., 2014), and the funding of researcher networks that aim to reduce environmental variability between laboratories, such as ZFIN and ZIRC. These and other initiatives have brought zebrafish to a new light as a translational model. However, unlike other animal models, longitudinal studies focused on behavior that could help to uncover developmental pharmacological mechanisms, developmental origins of health and disease, effects of epigenetics and other environmental processes, normal aging, as well as transgenerational effects are still scarce (Lange et al., 2013; Kalueff et al., 2014; Tal et al., 2020). In this review, we aimed to highlight the significance of such studies in light of pharmacological interventions and to examine possible adaptations in already established behavioral paradigms that would allow such longitudinal studies to be feasible throughout zebrafish life cycle. However, questions around this subject are left open.

First, while some of the studies presented here point to the feasibility of adapting tasks across ages, the face and construct validity of these strategies must be tested. Even more so given that other developmental stages such as the juvenile and old-age stages are less characterized than larvae and adults. Secondly, for some behavioral domains, such as cognition and sociability presented here, the adaptation of existing protocols is a challenging task. This is due mainly because of inconsistencies of results at early life stages, about when these behaviors start. To bridge this gap, a close observation of some points must be made. Early neural development in zebrafish is highly dependent on environmental factors such as temperature and housing density (Parichy et al., 2009). Data suggest that body length rather than age in days post-fertilization is a better indicator of the neural stage (Polverino et al., 2016; Yashina et al., 2019; Stednitz and Washbourne, 2020). Therefore, standardizing behavioral paradigms in zebrafish can also serve as a tool to find sensitive time points to assess different behavioral domains in this species. It is also important to note that while behavioral phenotypes are a complex product of CNS activity (Stewart et al., 2014), the effort to standardize behavioral paradigms must be coupled with the studies on the pharmacokinetics and pharmacodynamics of drugs and how these mechanisms develop along with the developing brain. Finally, it would be beneficial for the field if for the proposal and construction of new paradigms that the viability of its use across the developmental stages of zebrafish may be considered.
